# Factors That Determine the Outcomes of Surgical Versus Conservative Management in Achilles Tendon Ruptures: A Retrospective Cohort Study

**DOI:** 10.7759/cureus.74338

**Published:** 2024-11-24

**Authors:** Salim M Abduljawad, Yaser Almonla, Abdullah Bin Sahl, Rana Balvinder, Anand Pillai

**Affiliations:** 1 College of Medicine, Alfaisal University, Riyadh, SAU; 2 Trauma and Orthopaedics, Wythenshawe Hospital, Manchester University NHS Foundation Trust, Manchester, GBR; 3 Trauma and Orthopaedics, Royal College of Surgeons in Ireland, Dublin, IRL

**Keywords:** achilles tendon injury, achilles tendon repair, achilles tendon surgery, conservative management of the achilles tendon, conservative vs. surgical management, musculoskeletal rehabilitation, orthopaedic surgery, sports medicine, tendon rupture

## Abstract

Introduction: Achilles tendon rupture is the most commonly ruptured tendon in humans. Recent evidence suggests no significant differences in long-term functional outcomes between conservative and surgical management. Conservative treatment carries a higher risk of re-rupture, while surgical management presents risks such as wound infections and sural nerve damage. This retrospective cohort study aims to compare functional outcomes and patient-specific factors in conservative versus surgical management of Achilles tendon ruptures using the Achilles Tendon Total Rupture Score (ATRS).

Methods: Hospital electronic records from a major acute teaching hospital were reviewed to gather data on patients treated for Achilles tendon rupture. Patients were categorised into surgical or conservative treatment groups, and ATRSs were recorded at least one year post-injury. Statistical analysis, including the Wilcoxon rank sum and Welch t-tests, was used to compare ATRS outcomes between the groups. Outcomes were also stratified by age, sex, side of rupture, and tendon gap did not yield significant differences in outcomes. Notably, all female patients in this study were treated conservatively, precluding direct gender comparisons within the surgical treatment group. In addition, no consistent trends emerged regarding the side or specific location of the rupture.

Results: The final sample comprised 25 patients, excluding those with missing data and patients over 75 years old. No significant difference was observed in the overall ATRS between the surgical (mean 89.9) and conservative (mean 89.2) management (p = 0.662). However, older males (aged 51-75) demonstrated better outcomes with surgical management (mean ATRS 95.4) compared to conservative management (mean 86.2, p = 0.027). Younger males (aged 18-50) showed a trend toward better outcomes with conservative treatment (mean 91.9) compared to surgery (mean 80.7), although this was not statistically significant. Patients with larger tendon gaps (≥20 mm) had higher ATRSs regardless of treatment modality, but these findings did not reach statistical significance.

Conclusions: This study found no significant difference in long-term functional outcomes between conservative and surgical management of Achilles tendon ruptures. However, older males benefited more from surgery, while younger males showed a trend toward better outcomes with conservative treatment, but the decision to offer surgery should not be based on age alone. Better functional outcomes were found irrespective of the tendon gap in both groups. Further research with larger sample sizes is needed to validate these findings and guide patient-specific treatment decisions.

## Introduction

The Achilles tendon is the most commonly ruptured tendon in humans [1]. Recently, there has been a notable shift toward the conservative management of Achilles tendon ruptures, driven by numerous reports indicating no significant differences in functional outcomes between conservative and surgical interventions [2-6]. These findings are particularly relevant given the potential complications associated with surgical treatment. The literature highlights concerns regarding both approaches: conservative treatment is linked to higher re-rupture rates, while surgical intervention poses risks of complications, including wound infections and nerve damage.

Several studies have reported comparable functional outcomes and physical performance in patients following non-operative management and open surgical repair of Achilles tendon ruptures [2,3]. A systematic review and meta-analysis of 10 randomised controlled trials (RCTs) encompassing 944 patients and 19 cohort studies involving 14,918 patients further supported these findings [4]. This analysis revealed a heightened risk of re-rupture following non-operative treatment, while surgical management was associated with complications such as infections and nerve injuries. However, the authors noted significant variability in the size of the trials and the treatment and rehabilitation protocols employed across the studies.

Recent evidence suggests that accelerated functional rehabilitation, characterised by early weight-bearing and mobilisation, may reduce the risk of re-rupture in non-operative protocols [5,6]. Despite this, the literature remains divided, with some studies failing to demonstrate a clear reduction in re-rupture rates with accelerated rehabilitation [7-9]. In a large multicentre RCT comparing non-operative management, open surgical repair, and minimally invasive surgery (MIS) for acute Achilles tendon ruptures, Myrvold et al. [9] concluded that although surgery did not lead to better functional outcomes at 12 months, non-operative treatment carried a higher risk of tendon re-rupture.

Despite these findings, many centers have increasingly adopted non-operative approaches for managing most Achilles tendon ruptures. This retrospective cohort study aims to compare the functional outcomes of surgical and conservative treatments, contributing to the ongoing discourse on the optimal management of Achilles tendon ruptures.

## Materials and methods

Hospital electronic records from the orthopaedic department at Wythenshawe Hospital, a major acute teaching hospital in Wythenshawe, England, were accessed to collect data on patients who had been treated for Achilles tendon rupture. The information gathered included six key patient factors: age, sex, the side of the rupture, tendon gap, rupture location, and details regarding the management approach, whether conservative or surgical.

For conservative treatment, a non-operative approach was used. This typically involved immobilisation of the affected leg in a cast or brace with the foot placed in a plantarflexed position to facilitate tendon healing. After a period of immobilisation, patients transitioned to a walking boot and underwent a rehabilitation program aimed at gradually improving strength and range of motion. By contrast, the surgical treatment of Achilles tendon ruptures usually consisted of open or minimally invasive techniques. The torn tendon was surgically sutured to restore continuity, followed by immobilisation and a structured rehabilitation protocol similar to that used in conservative treatment.

The primary outcome measure in this study was the Achilles Tendon Total Rupture Score (ATRS), a widely validated and reliable instrument designed to evaluate patient-reported functionality following Achilles tendon rupture [10-12]. ATRSs were collected at 12-40 months post-surgery. Secondary outcome measures included the occurrence of complications of re-rupture and surgical wound infection or breakdown, which were recorded from the patients' files.

Analysis of the data was conducted with three objectives in mind: to compare the ATRS outcomes of conservative and surgical treatments, to analyse outcomes within the conservative treatment group, and to assess outcomes within the surgical treatment group, each stratified by the aforementioned patient demographics.

Due to the limited sample size presented, a pragmatic approach was necessary in analysing the patient factors. To accommodate this, six key factors were recorded for each patient alongside their ATRS data. These factors were grouped for analysis into equal-sized sub-groups for each factor. This utilised "high-low" splits based on the respective median values, as shown in Table 1.

**Table 1:** Groups divided according to the key factors

Factor	Sub-group (n patients)	Sub-group (n patients)
Age (years)	18-50 (12)	51-75 (13)
Tendon gap (mm)	<20 (11)	20+ (14)
Rupture location (cm)	<6 (13)	6+ (12)
Gender (F/M)	Female (4)	Male (21)
Tendon (L/R)	Left (12)	Right (13)
Management	Conservative (17)	Surgical (8)

Statistical analysis employed both non-parametric and parametric tests to compare ATRS outcomes between groups. The Wilcoxon rank sum test (also known as the Mann-Whitney U Test) was used to compare the distribution of ATRSs between the conservative and surgical groups without assuming normality in the data. In addition, the Welch t-test was employed, which assumes normal data distribution but does not require equal variances between the groups. Both tests were two-sided with a significance level set at 5% (α = 0.05). The p-values for both were reported to allow for a deeper interpretation of the results.

In assessing the distribution of ATRS data, the Shapiro-Wilk test was used to test the normality assumption. Although some tests indicated that the data could be normally distributed, histograms of the ATRSs often suggested a skew towards higher values, which could be attributed to the subjective nature of patient-reported outcomes. As a result, the Wilcoxon test results are presented as the primary finding, with the corresponding Welch test results included for comparison. All statistical analyses were performed using R version 4.3.1 statistical computing environment developed by the R Foundation for Statistical Computing in Vienna, Austria.

It is important to note that no female patients underwent surgical treatment, and as such, comparisons of ATRS outcomes between conservative and surgical management were limited to male patients only.

## Results

**Figure 1:**
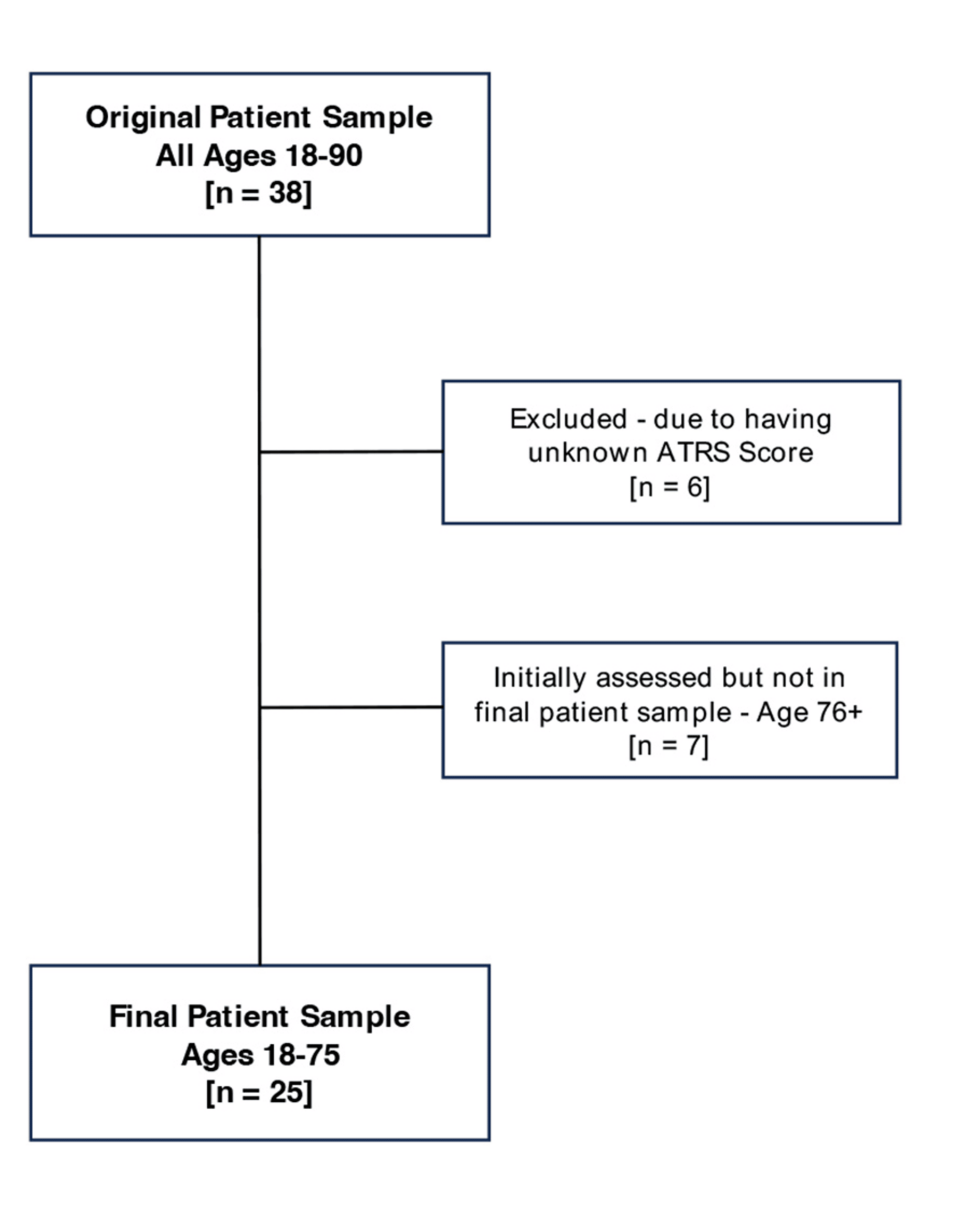
Process of selecting the patient cohort in the study. Summary data flow chart presented.

The initial sample comprised 38 patients. Six patients were excluded from the analysis due to missing ATRSs, and seven patients aged 76 years or older were excluded as out-of-scope for the study due to age-related confounding factors. This left a final sample of 25 patients for the analysis. This process is illustrated in the summary data flow chart (Figure 1), along with a comprehensive overview of the patient-level data, as shown in Table 2. As shown, six factors are recorded for each patient, along with their ATRS. Included in Appendix 1, but not the body of this article, is a summary of the ATRS outcomes for the excluded patients aged 76 as an additional analysis. Appendix 2 presents the histograms of the ATRSs to assess normality but suggests a skew toward higher values as aforementioned in the Materials and Methods section.

**Table 2:** Detailed patient-level data of the cohort that participated in this study. The total number of patients in the final cohort, excluding the last seven patients excluded aged 76+ also in the table, consisted of 25 patients as above.

Age (years)	Gender (M/F)	Tendon (R/L)	Rupture location (cm)	Tendon gap (mm)	Management (surgical / conservative)	ATRS (score 0-100)
Eight males surgically managed (ages 29-66); note: 0 females with surgery						
29	M	R	6.3	18	S	73
34	M	L	6.0	5	S	69
45	M	R	3.6	20	S	100
55	M	R	8.5	23	S	92
57	M	L	6.0	30	S	100
57	M	L	4.0	0	S	94
61	M	R	3.8	12	S	93
66	M	L	9.0	20	S	98
13 males conservatively managed (ages 34-75)						
34	M	L	5.0	20	C	100
37	M	R	2.9	22	C	98
38	M	R	5.0	17	C	65
43	M	R	6.0	20	C	94
44	M	L	6.0	23	C	97
45	M	L	5.3	18	C	100
47	M	R	5.5	10	C	89
57	M	L	8.3	25	C	94
61	M	R	6.7	2	C	84
67	M	L	6.0	15	C	90
69	M	R	4.5	16	C	75
72	M	R	8.0	20	C	84
75	M	L	1.1	51	C	90
Four females conservatively managed (ages 23-67)						
23	F	R	4.0	20	C	90
35	F	R	6.0	60	C	100
55	F	L	2.6	19	C	76
67	F	L	4.8	30	C	81
Seven patients with ages 76+ (4 males and 3 females; all conservatively managed) – excluded from the study analysis						
76	M	L	1.0	33	C	35
78	M	R	7.0	35	C	90
82	M	L	7.0	37	C	73
93	M	L	5.0	12	C	90
77	F	L	5.5	11	C	29
78	F	L	4.3	40	C	90
90	F	R	3.2	38	C	80

Outcomes for conservative versus surgical management 

**Figure 2:**
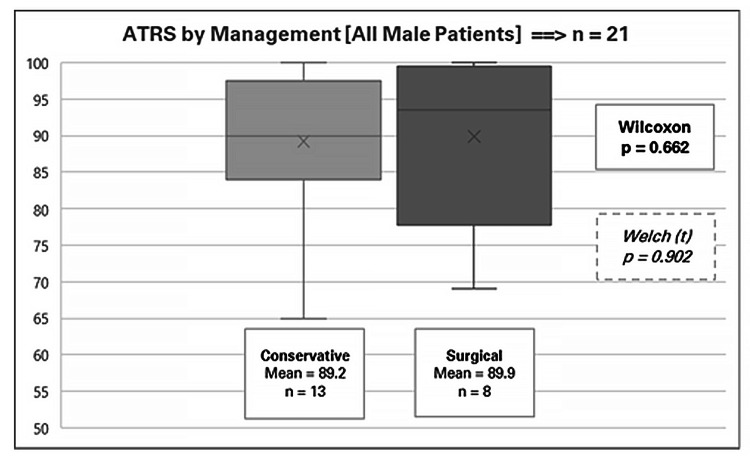
Achilles Tendon Total Rupture Score (ATRS) by management for all male patients

In this analysis, we focus exclusively on the male cohort (n = 21) as no female patients received surgical intervention. Of these, 13 patients underwent conservative management, and eight underwent surgical treatment. Specifically, the mean ATRS for surgical management was 89.9 (range 69-100), while the mean for conservative management was 89.2 (range 65-100). Statistical tests yielded a Wilcoxon p-value of 0.662 and a Welch t-test p-value of 0.902 (Figure 2). No statistically significant differences were observed in the mean ATRS between the two treatment modalities.

When further stratifying outcomes by patient factors (as detailed in Appendix 3), some notable variations in the mean ATRS emerged between the conservative and surgical groups. However, only one of these comparisons achieved statistical significance at the 5% level. Among the males aged 51-75, the mean ATRS for surgical management (95.4) was significantly higher than that for conservative treatment (86.2), with the Wilcoxon and Welch tests producing p-values of 0.027 and 0.020, respectively. Despite this significant finding, the small sample sizes warrant cautious interpretation.

An interesting trend emerged among younger males (aged 18-50) for whom the mean ATRS was lower following surgical management (80.7) compared to conservative management (91.9). However, this result did not reach statistical significance, likely due to the limited number of surgical patients in this age group (n = 3). If taken at face value, these findings might suggest that surgical intervention improves outcomes in older males but may be less advantageous - or even detrimental - for younger males. However, given the small sample sizes, these conclusions should be approached with caution.

Outcomes within conservative management stratified by patient factors

**Figure 3:**
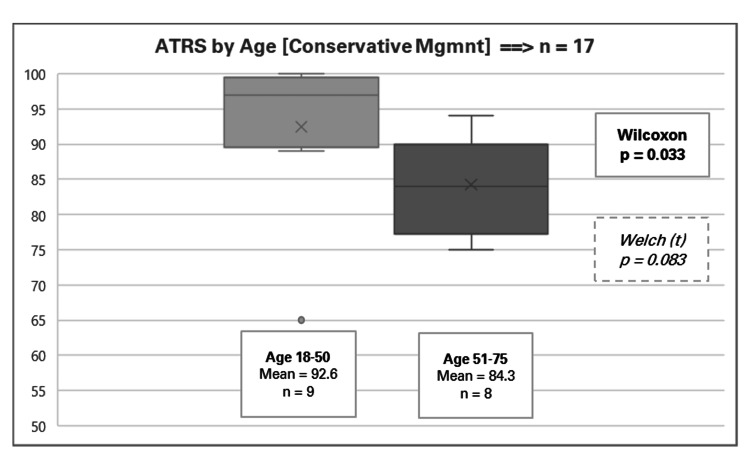
Achilles Tendon Total Rupture Score (ATRS) by age for conservative management

A total of 17 patients received conservative management (13 males and four females), with an overall mean ATRS of 88.6 (range 65-100). Appendix 4 presents the detailed results for conservative management, stratified by five patient factors. Among these, age was the only factor to reach statistical significance at the 5% level. The Wilcoxon p-value was 0.033 and a Welch t-test p-value was 0.083. As depicted in Figure 3, younger patients (aged 18-50) achieved higher mean ATRSs (92.6, range 65-100) compared to their older counterparts (aged 51-75) who had a mean score of 84.3 (range 75-94).

**Figure 4:**
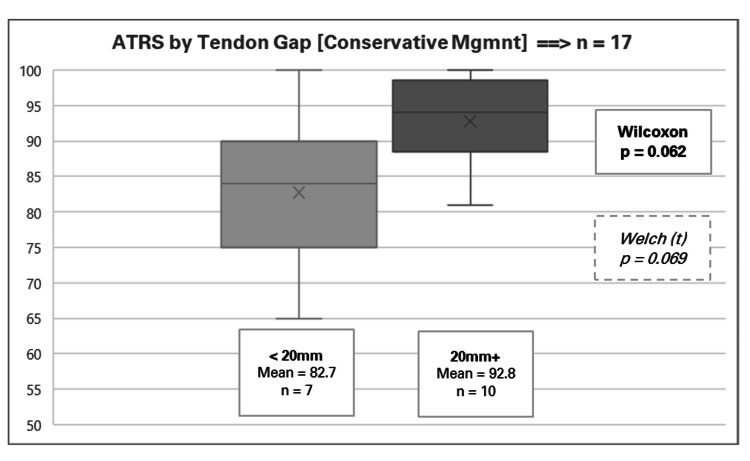
Achilles Tendon Total Rupture Score (ATRS) by tendon gap for conservative management

In addition, the factor of tendon gap approached significance, with a Wilcoxon p-value of 0.062 and a Welch p-value of 0.069. The data suggest that patients with a larger tendon gap had better outcomes (mean ATRS 92.8, range 81-100) than those with smaller gaps (mean ATRS 82.7, range 65-100), depicted in Figure 4. These findings may initially seem counterintuitive, but one potential explanation could be that patients with more severe injuries experience a greater sense of improvement in function and ATRSs post-treatment, resulting in higher subjective outcome scores. However, this is a speculative interpretation, and the result did not meet the threshold for statistical significance.

Outcomes within surgical management stratified by patient factors

**Figure 5:**
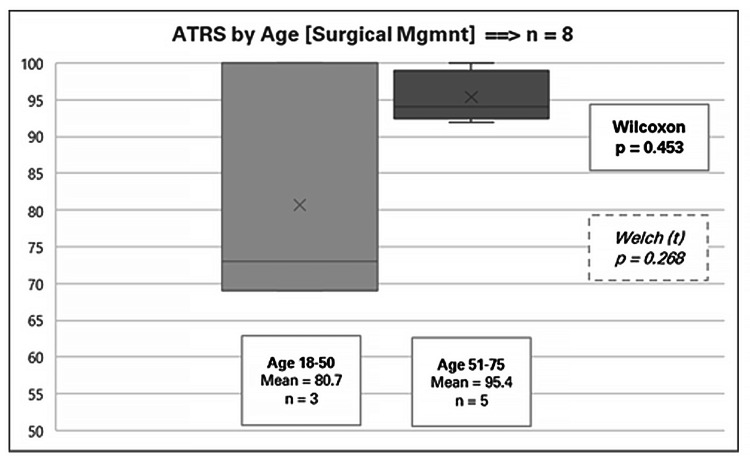
Achilles Tendon Total Rupture Score (ATRS) by age for surgical management

Eight male patients received surgical treatment, with an overall mean ATRS of 89.9 (range 69-100). Appendix 5 provides a detailed breakdown of the outcomes. None of the factors examined reached statistical significance at the 5% level, likely due to the small sample sizes involved. Nonetheless, some notable trends were observed. For instance, older males (mean ATRS 95.4) had higher outcome scores than younger males (mean ATRS 80.7. It can be argued that the small number of patients in each group (n = 5 and n = 3, respectively) suggests that these differences could be attributed to random variation rather than a true effect of age-related surgical outcomes (Figure 5).

**Figure 6:**
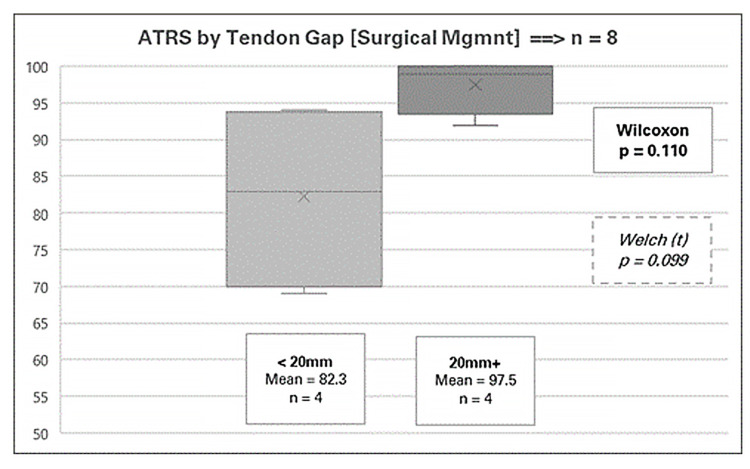
Achilles Tendon Total Rupture Score (ATRS) by tendon gap for surgical management

The factor close to achieving statistical significance was the tendon gap, with a Wilcoxon p-value of 0.110 and a Welch p-value of 0.099. The data indicated that patients with a larger tendon gap experienced better outcomes (mean ATRS 97.5, range 92-100) compared to those with smaller gaps (mean ATRS 82.3, range 69-94), as shown in Figure 6. This result parallels the trend observed in conservative management, where larger tendon gaps were also associated with higher ATRSs. As with the conservative cohort, this finding may be influenced by the patients' perception of relative improvement in function and ATRS from a more severe pre-treatment condition.

## Discussion

This retrospective cohort study compared functional outcomes between the surgical and conservative management of Achilles tendon ruptures, with particular attention to patient-specific factors. The primary finding that there is no statistically significant difference in the ATRS between the two treatment groups at one-year follow-up (p = 0.34) challenges the traditional notion that surgical intervention is inherently superior. This is consistent with emerging evidence that non-operative management using immobilisation, particularly when combined with early functional rehabilitation, can achieve outcomes comparable to surgical treatment [13]. Our results align with previous studies by Nilsson-Helander et al. [2], Olsson et al. [3], and Ochen et al. [4], all of which suggest that conservative treatment can be as effective as surgery in the long term.

However, while the overall ATRSs for conservative (89.2) and surgical (89.9) management were similar, nuances in the data reveal important clinical considerations. One key observation is the higher re-rupture rate in the conservatively treated group (14.3%) compared to the surgically treated group (2.9%). This finding is consistent with well-established risks of non-operative treatment [14]. On the other hand, surgical management was associated with a complication rate of 11.4%, primarily due to wound-related issues. This trade-off between the increased risk of re-rupture with conservative management and the potential complications associated with surgery highlights the complexity of deciding between these treatment options.

Our findings emphasise the importance of shared decision-making in the management of Achilles tendon ruptures. Treatment decisions should consider the individual patient's preferences, lifestyle, and risk tolerance. For example, younger, more active individuals may prioritise minimising re-rupture risk and thus opt for surgery. By contrast, older or less active patients may prefer conservative treatment to avoid the potential complications associated with surgery. This approach reflects the growing recognition that personalised treatment strategies, tailored to each patient’s unique circumstances, lead to better outcomes.

Age appeared to be a significant factor influencing outcomes, particularly in older males. Among males aged 51-75, those managed surgically had significantly better ATRS outcomes (mean 95.4) compared to those managed conservatively (mean 86.2) (Wilcoxon p = 0.027, Welch p = 0.020). This finding suggests that surgical intervention may provide enhanced functional recovery and satisfaction in older patients, possibly due to reduced biological healing potential with conservative management. On the contrary, among younger males (aged 18-50), conservative management was associated with better ATRS outcomes (mean 91.9) compared to surgery (mean 80.7), although this finding was not statistically significant due to the small sample size in the surgical group (n = 3). This may indicate that younger patients, with presumably better healing capacities, could benefit more from conservative management and avoid the risks associated with surgery.

The tendon gap was another notable factor in this study, revealing a somewhat counterintuitive trend. Patients with a larger tendon gap (≥20 mm) had better functional outcomes, regardless of the treatment modality, although these results were not statistically significant. This could reflect patients’ perception of greater improvement from a more severe baseline condition. However, more research is needed to fully understand the implications of tendon gap size on functional outcomes. In terms of treatment modality, Westin et al. found that surgical management was more effective for larger tendon gaps than conservative management [15]. 

Other patient factors, such as gender, side of rupture, and rupture location, did not yield significant differences in outcomes. It is important to note that all female patients in this study were treated conservatively, precluding direct gender comparisons within the surgical treatment group. In addition, no consistent trends emerged regarding the side or specific location of the rupture.

The trend toward an earlier return to sports in the surgical group, while not statistically significant, is worth considering. For athletes or individuals in physically demanding professions, a quicker return to full activity could be a decisive factor when choosing between surgical and conservative management. However, this potential benefit must be weighed against the increased risk of surgical complications, especially for younger or less injury-prone individuals.

This study has several limitations inherent to its retrospective design, including potential selection bias. Moreover, our reliance on patient-reported outcomes while utilising a validated instrument like the ATRS, introduces an element of subjectivity. Future prospective studies with larger sample sizes and standardised rehabilitation protocols are necessary to confirm our findings and refine treatment strategies. In addition, while this study focused on ATRS as a functional outcome measure, incorporating objective performance metrics and quality-of-life measures could provide a more comprehensive assessment of treatment efficacy.

Our findings emphasise the need for a paradigm shift in the management of Achilles tendon ruptures. Rather than applying a one-size-fits-all approach, clinicians should adopt personalised treatment plans that respect patient autonomy and preferences, incorporating both the potential benefits and risks of each treatment modality.

In conclusion, this study contributes to the growing body of evidence that non-operative treatment is a viable option for many patients with Achilles tendon ruptures. The lack of significant differences in long-term functional outcomes between surgical and conservative management suggests that the choice of treatment should be individualised. A comprehensive assessment of patient-specific factors, functional demands, and risk tolerance is essential for guiding treatment decisions. Shared decision-making informed by the latest evidence and tailored to the patient’s unique circumstances is crucial for optimising outcomes in Achilles tendon rupture management.

Further research should focus on identifying patient-specific predictors of treatment success and developing decision-making tools to facilitate informed choices. Additionally, refining rehabilitation protocols and exploring novel non-operative treatment modalities may improve outcomes and broaden the available options for patients.

## Conclusions

This study provides valuable insights into the comparative outcomes of surgical and conservative management for Achilles tendon ruptures, finding no significant difference in the overall mean ATRS between the two treatment modalities. However, specific patient factors, such as age and tendon gap size, may influence treatment effectiveness. Older males (aged 51-75) showed significantly better outcomes with surgical intervention (mean ATRS of 96.4) compared to conservative management (mean ATRS of 86.2), potentially due to reduced biological healing capacity mitigated by surgery. Conversely, younger males (aged 18-50) exhibited a trend toward better outcomes with conservative treatment (mean ATRS of 91.9 vs. 80.7 for surgery). In addition, patients with larger tendon gaps (≥20 mm) displayed better functional outcomes across both treatment types, although this finding did not reach statistical significance. These trends highlight the need for larger-scale studies to validate these observations and guide more patient-specific treatment recommendations.

## Figures and Tables

**Figure 7:**
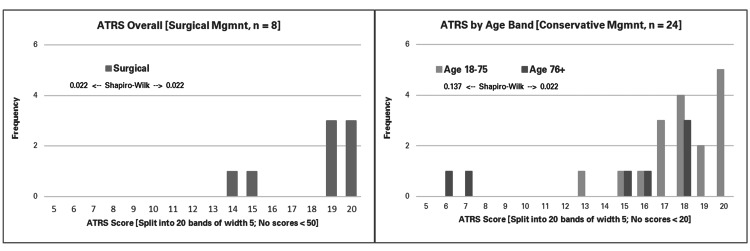
Achilles Tendon Total Rupture Score (ATRS) overall for surgical management on the left, ATRS by age for conservative management on the right

**Figure 8:**
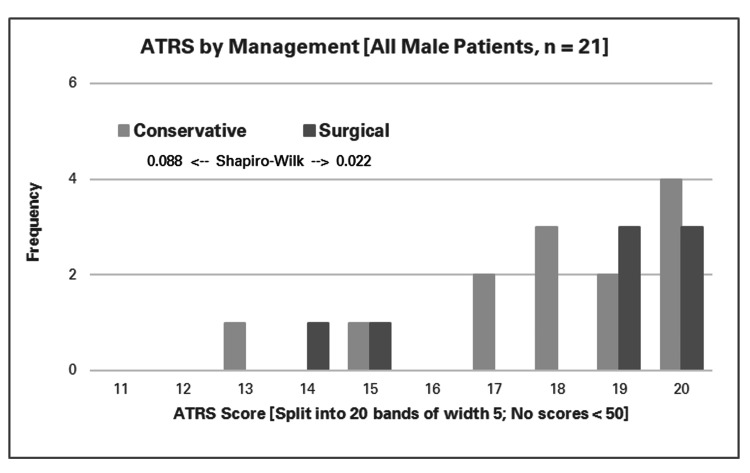
Achilles Tendon Total Rupture Score (ATRS) by management of all male patients

**Figure 9:**
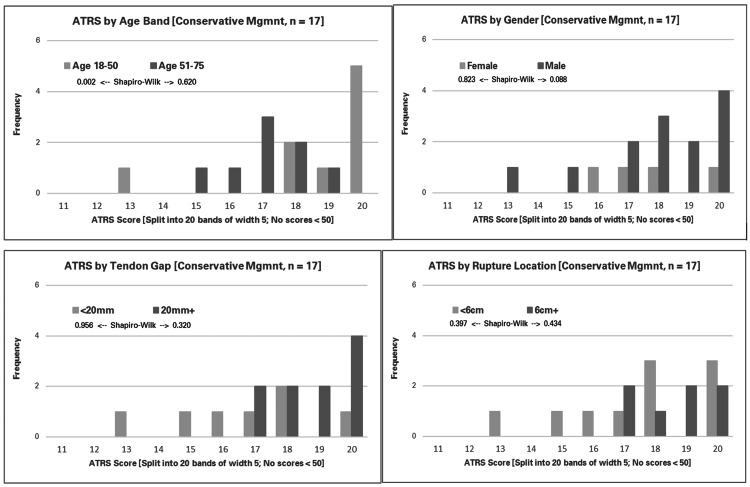
Achilles Tendon Total Rupture Score (ATRS) by age, gender, tendon gap, and rupture location for conservative management

**Figure 10:**
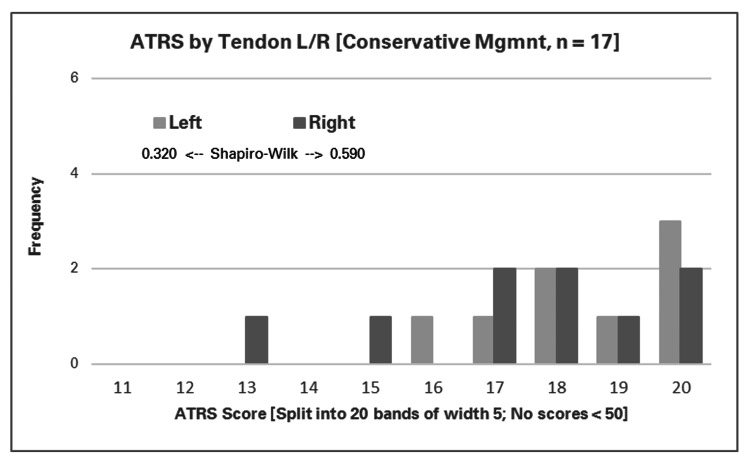
Achilles Tendon Total Rupture Score (ATRS) by tendon side L/R for conservative management

**Figure 11:**
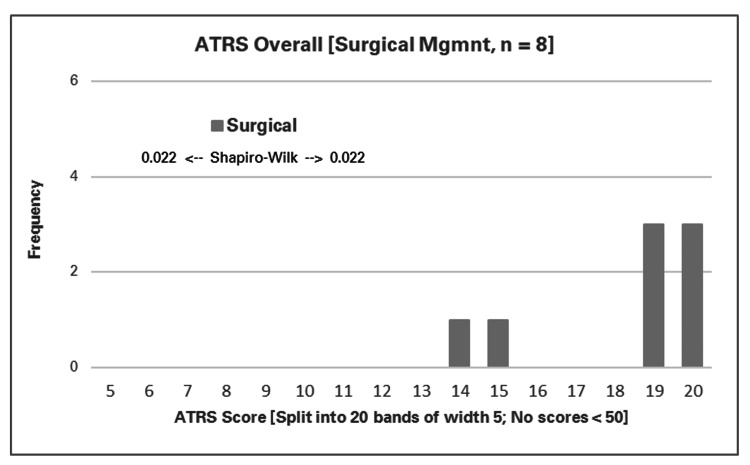
Achilles Tendon Total Rupture Score (ATRS) overall for surgical management

**Figure 12:**
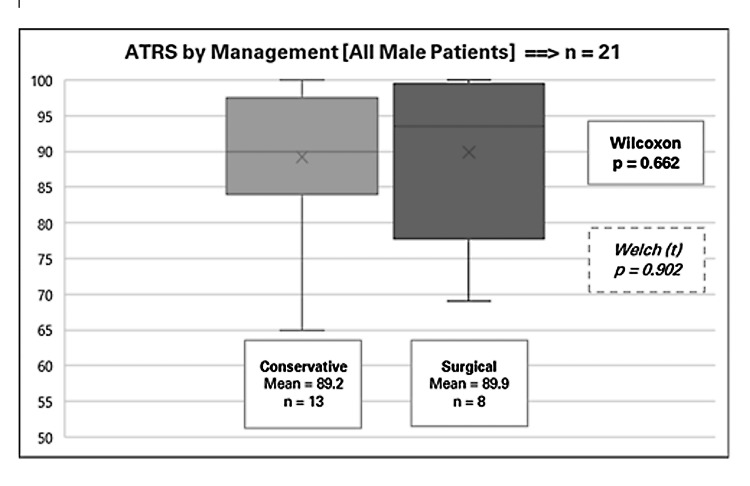
Achilles Tendon Total Rupture Score (ATRS) by management for all male patients

**Figure 13:**
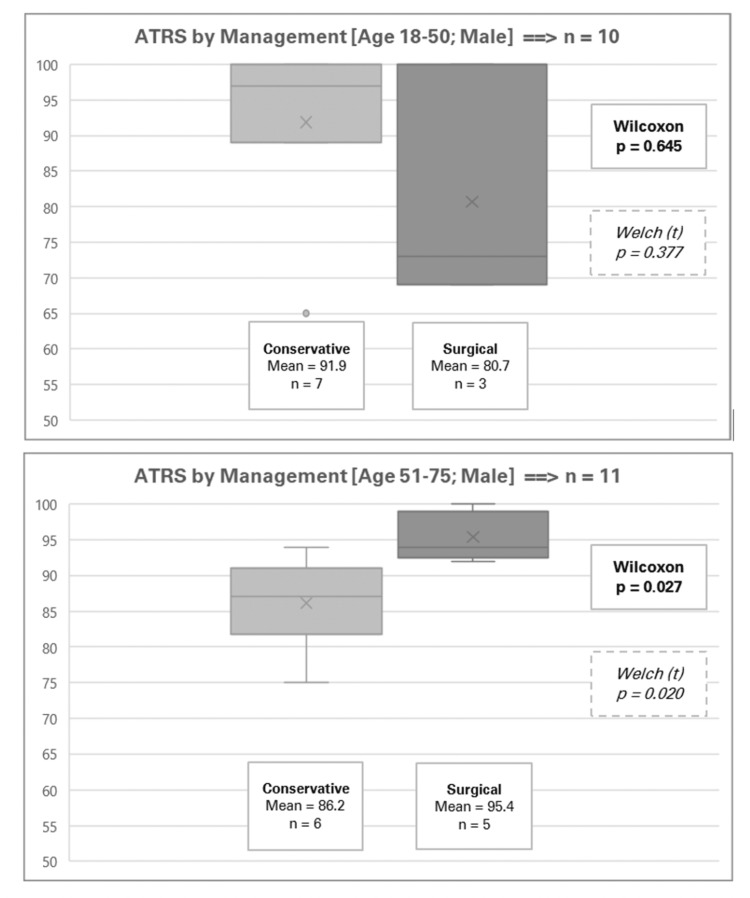
Achilles Tendon Total Rupture Score (ATRS) by management: ages 18-50 at the top of the image, ages 51-75 at the bottom of the image

**Figure 14:**
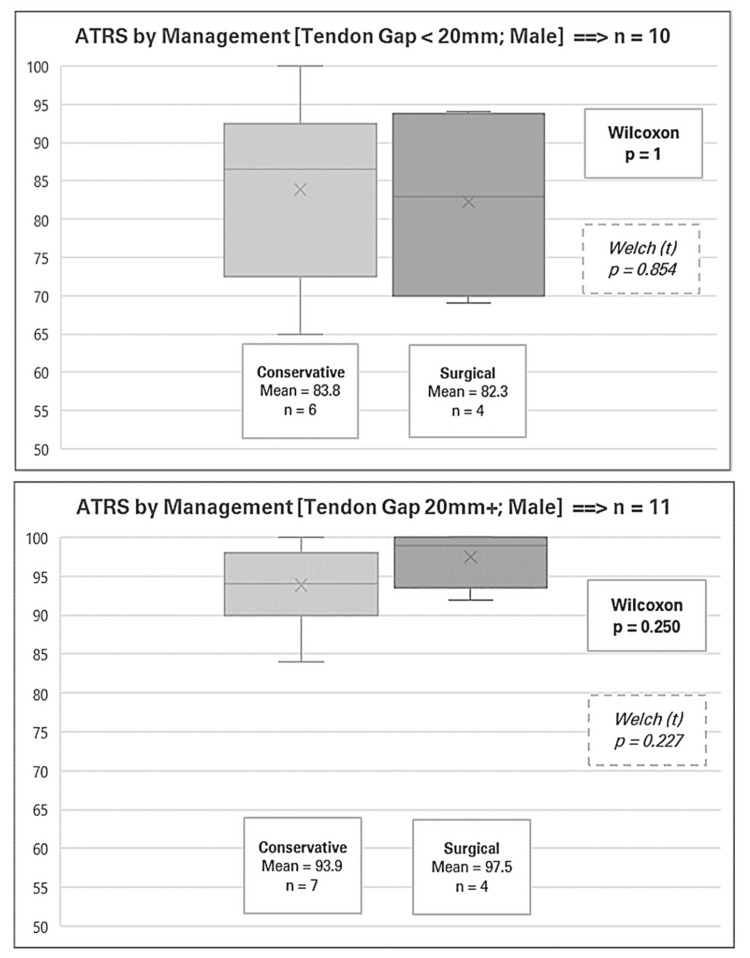
Achilles Tendon Total Rupture Score (ATRS) by management: tendon gap <20 mm at the top of the image, tendon gap 20 mm+ at the bottom of the image

**Figure 15:**
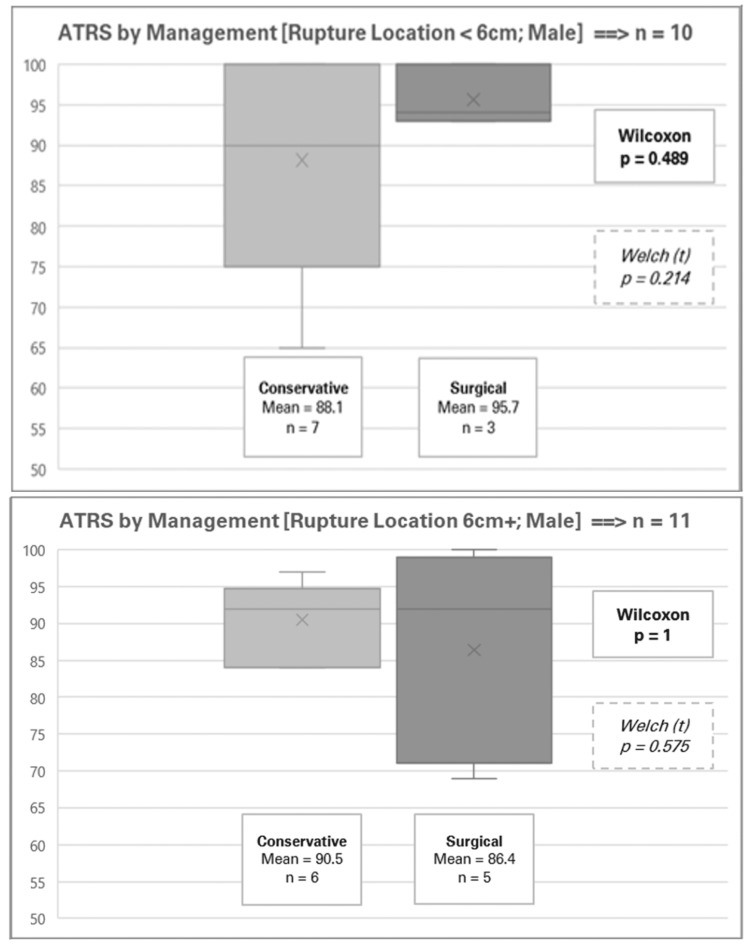
Achilles Tendon Total Rupture Score (ATRS) by management: rupture location <6 cm at the top of the image, 6 cm+ at the bottom of the image

**Figure 16:**
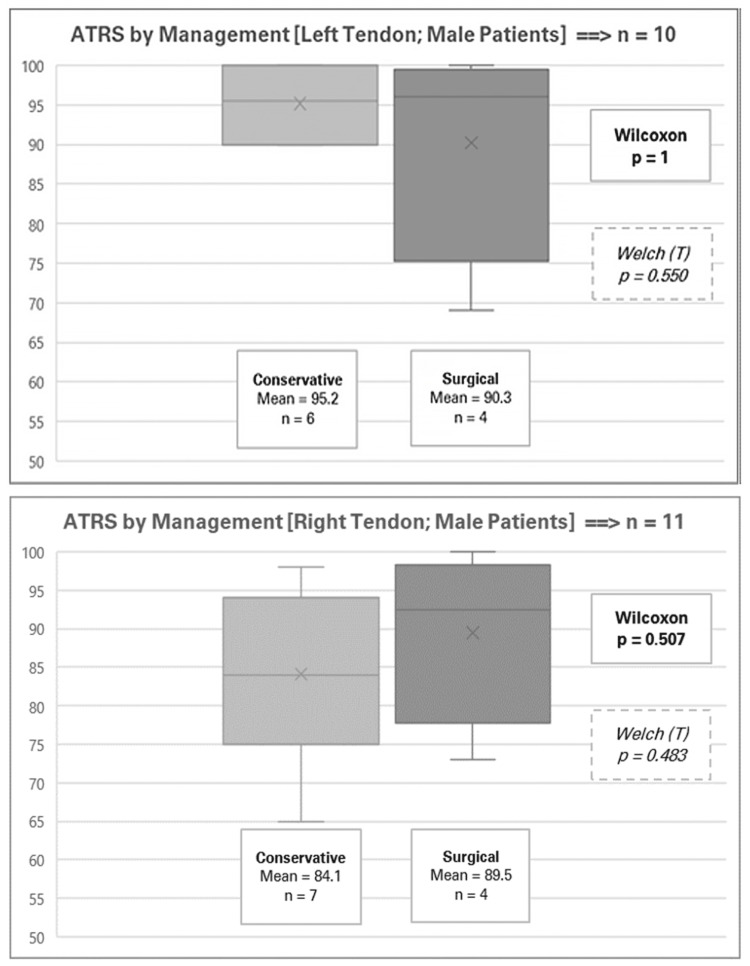
Achilles Tendon Total Rupture Score (ATRS) by management tendon side left side at the top of the image, right side at the bottom of the image

**Figure 17:**
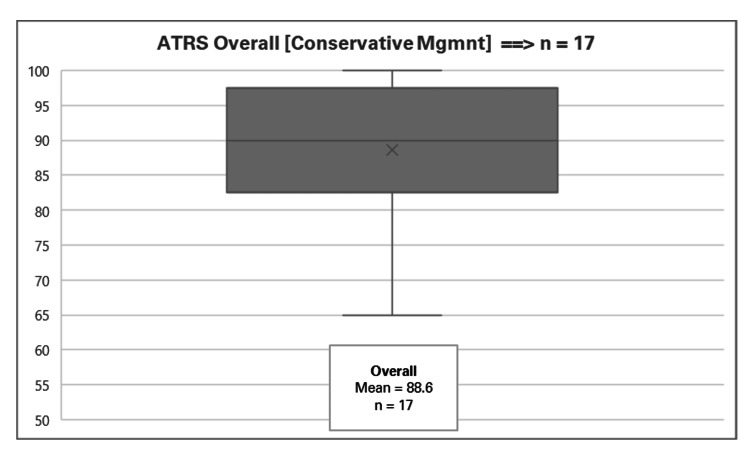
Achilles Tendon Total Rupture Score (ATRS) overall for conservative management

**Figure 18:**
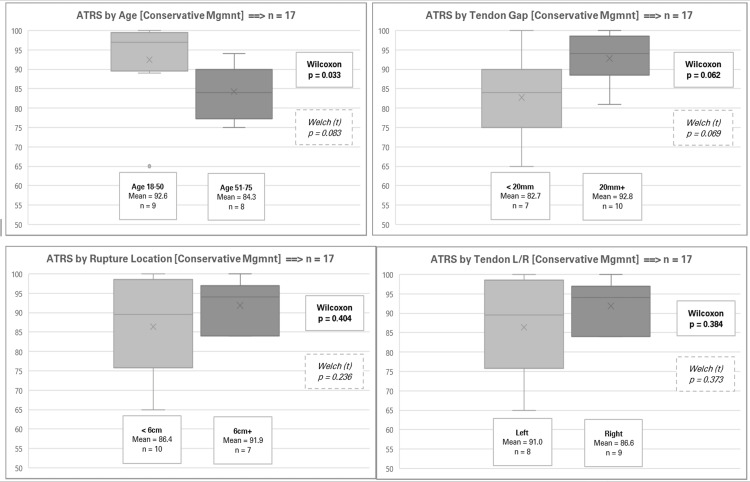
Achilles Tendon Total Rupture Score (ATRS) by age, tendon gap, rupture location, and tendon side for conservative management

**Figure 19:**
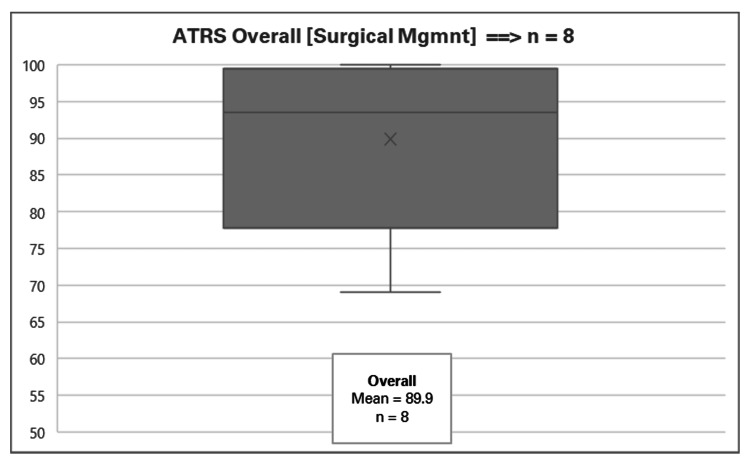
Achilles Tendon Total Rupture Score (ATRS) overall for surgical management

**Figure 20:**
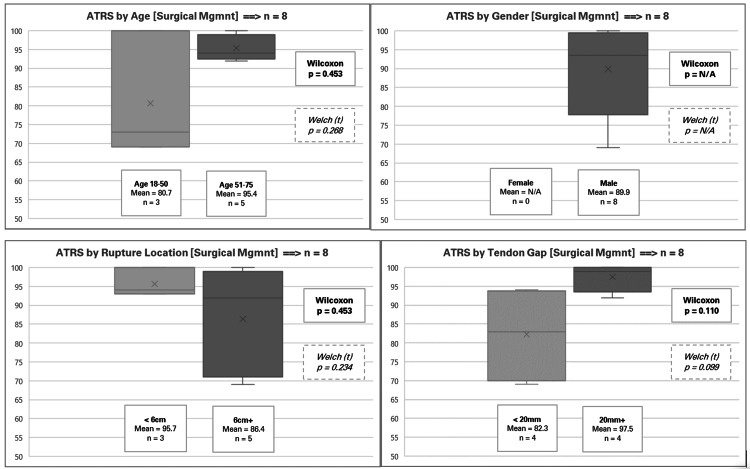
Achilles Tendon Total Rupture Score (ATRS) by age, gender, rupture location and tendon gap for surgical management

**Figure 21:**
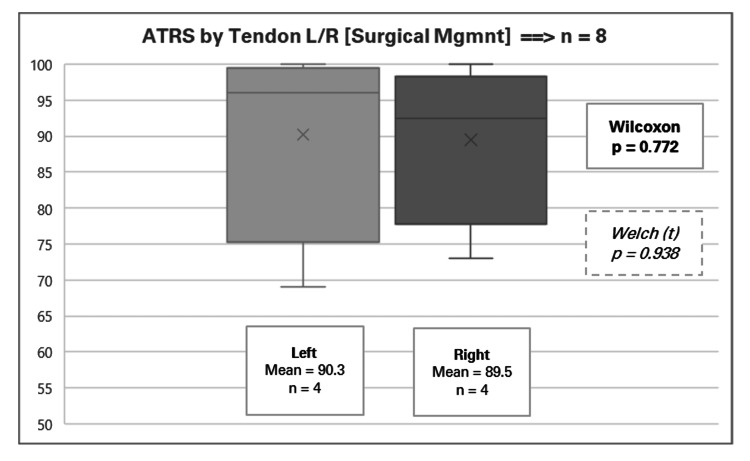
Achilles Tendon Total Rupture Score (ATRS) by tendon side for surgical management

## Appendices

The Appendix section presents additional analysis as figures and graphs: Appendix 1 (analysis of ATRS for the patients aged 76 and above excluded from the full analysis), Appendix 2 (histograms and Shapiro-Wilk tests for normality of various groups), Appendix 3 (conservative vs. surgical management), Appendix 4 (conservative management), and Appendix 5 (surgical management). 

Appendix 1: analysis of ATRS for patients aged 76 and above excluded from the full analysis

All patients aged 76 and over received conservative management (none had surgery). When comparing the ATRS outcomes for these older patients with their younger counterparts, we must restrict the younger “18-75” patients to those with conservative management (17 patients). 

The results for this are shown below. Patients aged 76+ had a lower mean ATRS (69.6) than those aged 18-75 (88.6). Regarding the Wilcoxon test, this result is very nearly (but not quite) significant at the 5% level (p = 0.059).

Figure 7 shows the boxplot and test results. 

Appendix 2: histograms and Shapiro-Wilk tests for normality of various groups

Note that ATRS was split into 20 groups of width 5 to plot histograms. Hence, in the charts below, a value of “20” on the x-axis relates to an ATRS of 96-100, 19 relates to 91-95, and so on. 

As mentioned earlier, when looking visually we can see that most of the groups analysed show a distribution skewed (to some extent or other) towards higher-end scores.  Notwithstanding the various Shapiro-Wilk tests undertaken (where the null hypothesis is often accepted), the assumption of normally distributed data is questionable, given the results in the charts below.

Figure 8 shows the outcomes for conservative versus surgical management.

Figures 9-10 show outcomes within conservative management.

Figure 11 shows outcomes within surgical management.

Appendix 3:  Boxplots and test results (conservative vs. surgical management)

This is presented in Figures 12-16. 

Appendix 4: boxplots and test results (conservative management)

This is presented in Figures 17-18. 

Appendix 5: boxplots and test results (surgical management)

This is presented in Figures 19-21.

## References

[1] Shamrock AG (2024). Achilles tendon rupture. StatPearls [Internet].

[2] Nilsson-Helander K, Silbernagel KG, Thomeé R, Faxén E, Olsson N, Eriksson BI, Karlsson J (2010). Acute achilles tendon rupture: a randomized, controlled study comparing surgical and nonsurgical treatments using validated outcome measures. Am J Sports Med.

[3] Olsson N, Silbernagel KG, Eriksson BI, Sansone M, Brorsson A, Nilsson-Helander K, Karlsson J (2013). Stable surgical repair with accelerated rehabilitation versus nonsurgical treatment for acute Achilles tendon ruptures: a randomized controlled study. Am J Sports Med.

[4] Ochen Y, Beks RB, van Heijl M (2019). Operative treatment versus nonoperative treatment of Achilles tendon ruptures: systematic review and meta-analysis. BMJ.

[5] Twaddle BC, Poon P (2007). Early motion for Achilles tendon ruptures: is surgery important? A randomized, prospective study. Am J Sports Med.

[6] Soroceanu A, Sidhwa F, Aarabi S, Kaufman A, Glazebrook M (2012). Surgical versus nonsurgical treatment of acute Achilles tendon rupture: a meta-analysis of randomized trials. J Bone Joint Surg Am.

[7] Barfod KW, Hansen MS, Hölmich P, Kristensen MT, Troelsen A (2020). Efficacy of early controlled motion of the ankle compared with immobilisation in non-operative treatment of patients with an acute Achilles tendon rupture: an assessor-blinded, randomised controlled trial. Br J Sports Med.

[8] Costa ML, Achten J, Marian IR (2020). Plaster cast versus functional brace for non-surgical treatment of Achilles tendon rupture (UKSTAR): a multicentre randomised controlled trial and economic evaluation. Lancet.

[9] Myhrvold SB, Brouwer EF, Andresen TK (2022). Nonoperative or surgical treatment of acute Achilles' tendon rupture. N Engl J Med.

[10] Spennacchio P, Vascellari A, Cucchi D, Canata GL, Randelli P (2016). Outcome evaluation after Achilles tendon ruptures. A review of the literature. Joints.

[11] Nilsson-Helander K, Thomeé R, Silbernagel KG, Thomeé P, Faxén E, Eriksson BI, Karlsson J (2007). The Achilles Tendon Total Rupture Score (ATRS): development and validation. Am J Sports Med.

[12] Kearney RS, Achten J, Lamb SE, Parsons N, Costa ML (2012). The Achilles tendon total rupture score: a study of responsiveness, internal consistency and convergent validity on patients with acute Achilles tendon ruptures. Health Qual Life Outcomes.

[13] van der Eng DM, Schepers T, Goslings JC, Schep NW (2013). Rerupture rate after early weightbearing in operative versus conservative treatment of Achilles tendon ruptures: a meta-analysis. J Foot Ankle Surg.

[14] Deng H, Cheng X, Yang Y (2023). Rerupture outcome of conservative versus open repair versus minimally invasive repair of acute Achilles tendon ruptures: A systematic review and meta-analysis. PLoS One.

[15] Westin O, Nilsson Helander K, Grävare Silbernagel K, Möller M, Kälebo P, Karlsson J (2016). Acute ultrasonography investigation to predict reruptures and outcomes in patients with an Achilles tendon rupture. Orthop J Sports Med.

